# Bone responsiveness to parathyroid hormone is negatively associated with parathyroid hormone-lowering drug use in patients undergoing hemodialysis: a cross-sectional study

**DOI:** 10.1186/s12882-021-02482-z

**Published:** 2021-08-09

**Authors:** Naoto Tominaga, Tomoki Yonaha, Masayuki Yamanouchi, Hirofumi Sumi, Yasuhiro Taki, Yugo Shibagaki, Kazuhiro Shiizaki, Shozo Yano

**Affiliations:** 1 Division of Nephrology and Hypertension, Department of Internal Medicine, St. Marianna University School of Medicine, 2-16-1, Sugao, Miyamae-ku, Kawasaki, Kanagawa 216-8511 Japan; 2 Division of Nephrology and Hypertension, Kawasaki Municipal Tama Hospital, Kawasaki, Kanagawa Japan; 3 Nephrology and Dialysis Center, Yohana Medical Clinic, Ishigaki, Okinawa Japan; 4 Nephrology Center, Toranomon Hospital Kajigaya, Kawasaki, Kanagawa Japan; 5 Department of Nephrology, Yurina Medical Park, Nogi, Tochigi Japan; 6 Department of Laboratory Medicine, Shimane University Faculty of Medicine, Izumo, Shimane Japan

**Keywords:** Hemodialysis, Parathyroid hormone, Tartrate-resistant acid phosphatase-5b, Calcium-sensing receptor agonists, Vitamin D receptor activators

## Abstract

**Background:**

Parathyroid hormone (PTH) acts on bone to indirectly increase the number and activity of osteoclasts. Thus, PTH has a stimulatory effect on bone resorption and upregulates bone turnover. However, the responsiveness of bone to PTH varies widely among patients receiving dialysis. In fact, relative to the serum PTH level, the level of serum tartrate-resistant acid phosphatase-5b (TRACP-5b), a bone resorption marker derived from osteoclasts, varies as well. This study aimed to examine factors related to bone responsiveness to PTH in patients undergoing chronic hemodialysis (HD).

**Methods:**

This study included patients receiving chronic HD in Kawasaki Municipal Tama Hospital (Kanagawa, Japan) and Yonaha Medical Clinic (Okinawa, Japan) and excluded patients who received HD for less than 6 months, those who received a combination of HD and peritoneal dialysis, and those who had cancer bone metastases or myeloma. The TRACP-5b/intact PTH (iPTH) ratio was created as an index of bone responsiveness to PTH, categorized into tertiles (low, medium, and high), and a cross-sectional study was conducted. *P* < 0.05 indicated statistically significant differences.

**Results:**

One hundred and six patients were analyzed. Age (*P* = 0.010), body mass index (BMI) (*P* = 0.003), use of calcium-sensing receptor (CaSR) agonists (*P* = 0.008), use of vitamin D receptor activators (VDRAs) (*P* = 0.012), plasma iPTH level (*P* < 0.001), serum 1,25(OH)_2_D level (*P* = 0.003), and serum TRACP-5b level (P < 0.001) were significantly different among the three categories. In the single linear regression analysis, age (*P* = 0.016), corrected serum calcium level (*P* = 0.007), and ln [1,25(OH)_2_D] (*P* = 0.044) showed a significant positive correlation with ln [TRACP-5b/iPTH], whereas BMI (*P* = 0.026), use of CaSR agonists (*P* = 0.001), use of VDRAs (*P* = 0.009), and serum phosphorus level (*P* = 0.018) showed a significant negative correlation. Upon conducting multiple linear regression analysis incorporating significant variables in the single linear regression analysis, a significant negative correlation was observed between the TRACP-5b/iPTH ratio and intravenous administration of a CaSR agonist (etelcalcetide) and/or a VDRA (calcitriol or maxacalcitol) in all the adjusted models.

**Conclusions:**

Bone responsiveness to PTH is negatively correlated with the intravenous administration of a CaSR agonist and/or a VDRA in patients undergoing chronic HD.

## Background

High bone turnover is the main pathophysiology of secondary hyperparathyroidism (SHPT) in patients undergoing chronic hemodialysis (HD) and leads to a high risk of fracture [1]. A report from the Dialysis Outcomes and Practice Patterns Study showed that the incidence of fractures was significantly higher in patients on chronic HD than that in the general population, with chronic HD patients showing a 3.7-fold higher unadjusted relative risk of death [2]. Parathyroid hormone (PTH) is one of the promoting factors for bone turnover. However, bone responsiveness to PTH varies from individual to individual. For example, African Americans show lower bone responsiveness to PTH than Caucasians; therefore, African Americans may require a higher PTH level to maintain the same degree of bone turnover as Caucasians [3].

PTH stimulates osteoblasts to secrete receptor activator of nuclear factor-kappa B ligand (RANKL), which then stimulates the osteoclast precursors to mature [4]. Mature osteoclasts produce tartrate-resistant acid phosphatase 5b (TRACP-5b), which is released into the circulation as a specific biomarker of osteoclast number and activity [5]. PTH levels can now be controlled through treatment of SHPT. Particularly, calcium-sensing receptor (CaSR) agonists and vitamin D receptor activators (VDRAs) are commonly used to control the high-turnover bone status in patients on chronic HD [6]. However, the association between the use of a CaSR agonist and/or a VDRA and bone responsiveness to PTH has not been reported in a population undergoing dialysis.

In the present study, we created the TRACP-5b/intact PTH (iPTH) ratio as an index of bone responsiveness to PTH, categorized into tertiles (low, medium, and high), and aimed to compare clinical and laboratory factors between the low, medium, and high TRACP-5b/iPTH ratio groups of patients undergoing chronic HD. We further aimed to examine the association between the use of a CaSR agonist and/or a VDRA and the TRACP-5b/iPTH ratio in outpatients undergoing chronic HD.

## Methods

### Patients and materials

Data for the present study were obtained from the pooled patient electronic health record system of the Kawasaki Municipal Tama Hospital in Kanagawa and Yonaha Medical Clinic in Okinawa. We retrospectively reviewed clinical and laboratory data from outpatients who had received chronic HD from April 2018 to September 2018. This study was conducted in accordance with the Declaration of Helsinki and was approved by the Institutional Committee on Human Research of St. Marianna University School of Medicine [the Institutional Review Board (IRB) at St. Marianna University School of Medicine (IRB approval number: 4291)]. The requirement for informed consent was waived due to the de-identified nature of the analyses.

### Study design and procedures

This retrospective cross-sectional study was designed to investigate the association between CaSR agonist and/or VDRA use (as a binary variable) and the TRACP-5b/iPTH ratio (as a continuous variable) among patients undergoing chronic HD. Patients who had been undergoing chronic HD for less than 6 months and those undergoing combined HD and peritoneal dialysis were excluded. Patients with bone metastases and/or multiple myeloma, which have potential effects on bone responsiveness to PTH, were also excluded.

We utilized the means of 11 consecutive values for serum albumin (Alb), calcium (Ca), and phosphorus (P) levels measured in the blood twice per month as well as the means of six consecutive values for the plasma intact parathyroid hormone (iPTH) level measured once per month prior to obtaining serum bone-specific alkaline phosphatase (BAP), TRACP-5b, 25-hydroxyvitamin D [25(OH)D], and 1,25-dihydroxycholecalciferol [1,25(OH)_2_D] level data as of September 2018. We used Payne’s formula to correct the serum Ca (cCa) level based on the serum Alb level [7]. The serum BAP level was measured using the chemiluminescent enzyme immunoassay (CLEIA) (LSI Medience Corporation, Chiyoda-ku, Tokyo, Japan), the serum TRACP-5b level was measured using the enzyme immunoassay (EIA) (LSI Medience Corporation), the plasma iPTH level was measured using the electrochemiluminescence immunoassay (ECLIA) (LSI Medience Corporation), the serum 25(OH)D level was measured using the chemiluminescence immunoassay (CLIA) (LSI Medience Corporation), and the serum 1,25(OH)_2_D level was measured using double-antibody radioimmunoassay (RIA) (LSI Medience Corporation). All blood tests were performed prior to HD, in the first HD session of the week. Patient exposure to clinical variables of interest was defined by data regarding duration of chronic HD, type of anticoagulant used during HD, disease diagnosis (i.e., diabetes, ischemic heart disease, or cerebrovascular disease), drug prescription [i.e., CaSR agonist (cinacalcet/evocalcet, etelcalcetide), VDRA (alfacalcidol/falecalcitriol, calcitriol, maxacalcitol), P binder (Ca containing, Non-Ca containing, both), steroid, β-blocker, statin, warfarin, or bisphosphonate], or history of fracture.

A recent study reported the non-inferiority of evocalcet to cinacalcet in suppressing the iPTH level by acting as an oral allosteric modulator of CaSR [8]; therefore, patients on evocalcet were grouped with the patients on cinacalcet. Etelcalcetide is an intravenous formula of a direct activator of CaSR; therefore, it was considered in a separate group although the number of patients using the drug was small. A patient receiving falecalcitriol was grouped with the patients receiving alfacalcidol, since both drugs are oral VDRAs. Data regarding the ankle-brachial index (ABI) were used, as peripheral artery disease is associated with low bone turnover in prevalent nondiabetic patients with end-stage renal disease (ESRD) [9]. The body mass index (BMI) was calculated by dividing weight (kg) by height squared (m^2^).

### Statistical analysis

All data were summarized using descriptive statistics, such as median, interquartile range (IQR), frequency, and percentage. For continuous variables, the Kruskal-Wallis test was used to assess the significance of differences in patient characteristics among the three TRACP-5b/iPTH ratio categories (low, medium, and high). For discrete variables, the chi-square test was used to compare data among the three groups. Single and multiple linear regression analyses were used to examine factors affecting the TRACP-5b/iPTH ratio as of September 2018. A multiple linear regression analysis was performed with adjustment for age, sex, BMI, and serum Alb level using variables identified as significant in the single linear regression analysis. In the single and multiple linear regression analyses, values of plasma iPTH, serum 25(OH)D, 1,25(OH)_2_D, BAP and TRACP-5b levels and the TRACP-5b/iPTH ratio were transformed to natural logarithms (ln) for their skewed distribution. Statistical analyses were performed using Stata version 14 (StataCorp LP, College Station, TX, USA). Statistical significance was defined at *P* < 0.05.

## Results

### Patient characteristics

Median patient age, BMI, and duration of chronic HD were 68 years, 21.5 kg/m^2^, and 7.9 years, respectively. The median serum Alb, cCa, P, BAP, and TRACP-5b levels were 3.6 g/dL, 9.1 mg/dL, 5.4 mg/dL, 14.2 μg/L, and 559 mU/dL, respectively. The median plasma iPTH level was 199.1 pg/mL. Among the 106 patients included, 45 were women (42.5%), 38 had diabetes (type 2 diabetes in all cases) (35.8%), 61 had been prescribed CaSR agonist (57.5%), and 97 had been prescribed VDRA (91.5%). The causes of ESRD included diabetic nephropathy in 34 cases (32.1%), nephrosclerosis in 25 cases (23.6%), glomerulonephritis in 28 cases (26.4%), and other causes in 19 cases (17.9%) (Table 1).

**Table 1:** Patient characteristics

	*N* = 106
Age, years	68 [60. 75]
Female sex, n (%)	45 (42.5)
BMI, kg/m^2^	21.5 [19.6, 24.3]
Chronic HD vintage, years	7.9 [2.9, 12.3]
Anticoagulant during HD, n (%)	106 (100)
None	0 (0)
Heparin	90 (84.9)
LMWH	10 (9.4)
Nafamostat	6 (5.7)
Cause of ESRD, n (%)	106 (100)
Diabetic nephropathy	34 (32.1)
Nephrosclerosis	25 (23.6)
Glomerulonephritis	28 (26.4)
Others	19 (17.9)
Diabetes, n (%)	38 (35.8)
Ischemic heart disease, n (%)	27 (25.5)
Cerebrovascular disease, n (%)	18 (17.0)
CaSR agonist use, n (%)	61 (57.5)
None	45 (42.5)
Cinacalcet/ Evocalcet	55 (51.9)
Etelcalcetide (i.v.)	6 (5.7)
VDRA use, n (%)	97 (91.5)
None	9 (8.5)
Alfacalcidol/ Falecalcitriol	48 (45.3)
Calcitriol (i.v.)	32 (30.2)
Maxacalcitol (i.v.)	17 (16.0)
P binder use, n (%)	90 (84.9)
None	16 (15.1)
Ca containing	12 (11.3)
Non-Ca containing	55 (51.9)
Both	23 (21.7)
Steroid use history, n (%)	13 (12.3)
β blocker use, n (%)	48, (45.3)
Statin use, n (%)	28 (26.4)
Warfarin use, n (%)	7 (6.6)
Bisphosphonate use, n (%)	14 (13.2)
Fracture history, n (%)	15 (14.2)
Ankle-brachial index	1.10 [1.03, 1.18]
Alb, g/dL	3.6 [3.4, 3.8]
cCa, mg/dL	9.1 [8.5, 9.4]
P, mg/dL	5.4 [4.5, 6.0]
iPTH, pg/mL	199.1 [119.0, 277.2]
25(OH)D, ng/mL	14.3 [11.0, 18.9]
1, 25(OH)_2_D, pg/mL	13 [8, 19]
BAP, μg/L	14.2 [10.8, 18.3]
TRACP-5b, mU/dL	559 [339, 833]

*Abbreviations*: *Alb* Serum albumin, *BAP* Bone-specific alkaline phosphatase, *BMI* Body mass index, *CaSR* Calcium-sensing receptor, *cCa* Corrected Ca, *ESRD* End-stage renal disease, *HD* Hemodialysis, *iPTH* Intact parathyroid hormone, *i.v.* Intravenous, *LMWH* Low-molecular-weight heparin, *P* Phosphorus, *TRACP-5b* Tartrate-resistant acid phosphatase-5b, *VDRA* Vitamin D receptor activator

Values are expressed as the median [interquartile range (IQR)] or as frequencies and percentages [n (%)]

### Comparison of the three TRACP-5/iPTH ratio categories (low, medium, and high)

**Fig. 1:**
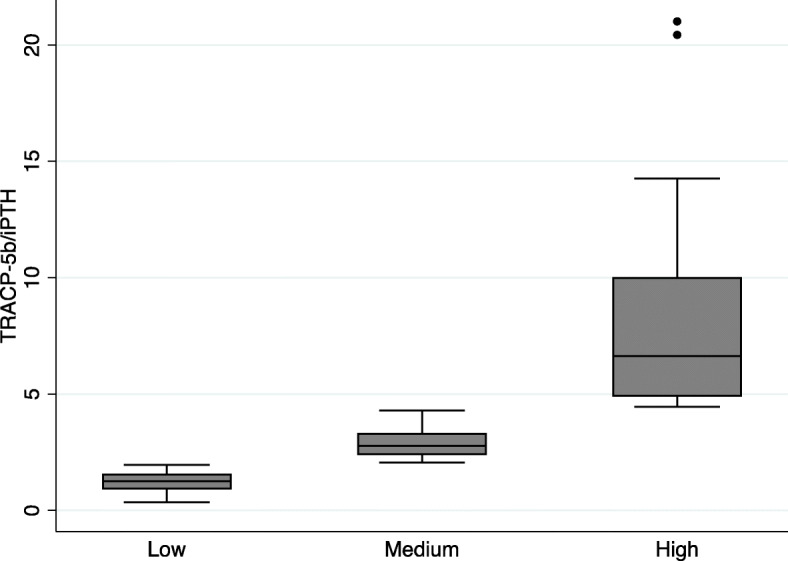
Box plots of differences in the TRACP-5b/iPTH ratio among the three categories of patients. Abbreviations: iPTH, intact parathyroid hormone; TRACP-5b, tartrate-resistant acid phosphatase-5b

We compared patient attributes and laboratory data among the low, medium, and high TRACP-5/iPTH ratio categories (Table 2, Fig. 1).

**Table 2:** Comparison of the TRACP-5b/iPTH ratio among the three categories (low, medium, and high)

	TRACP-5b/iPTH ratio			***P*** value
	Low (Quartile 1)	Medium (Quartile 2)	High (Quartile 3)	
	< 2.06	≧2.06, < 4.46	4.46≦	
N	36	35	35	–
Age, years	65 [54, 72]	68 [63, 75]	69 [64, 76]	0.010
Female sex, n	13	15	17	0.568
BMI, kg/m^2^	21.9 [20.6, 24.6]	23.0 [20.1, 27.1]	20.1 [18.4, 21.8]	0.003
Chronic HD vintage, years	6.8 [3.6, 13.5]	8.7 [3.0, 12.3]	8.0 [2.5, 12.0]	0.730
Anticoagulant during HD, n (%)				
Heparin	30 (83.3)	31 (88.6)	29 (82.9)	0.299
LMWH	4 (11.1)	4 (11.4)	2 (5.7)	
Nafamostat	2 (5.6)	0 (0)	4 (11.4)	
Cause of ESRD, n				
Diabetic nephropathy	12	9	13	0.840
Nephrosclerosis	9	9	7	
Glomerulonephritis	10	11	7	
Others	5	6	8	
Diabetes, n	15	10	13	0.506
Ischemic heart disease, n	10	9	8	0.892
Cerebrovascular disease, n	9	4	5	0.274
CaSR agonist use, n (%)	26 (72.2)	22 (62.9)	13 (37.1)	0.008
None	10 (27.8)	13 (37.1)	22 (62.9)	0.001
Cinacalcet/ Evocalcet	20 (55.6)	22 (62.9)	13 (37.1)	
Etelcalcetide (i.v.)	6 (16.7)	0 (0)	0 (0)	
VDRA use, n (%)	35 (97.2)	34 (97.1)	28 (80.0)	0.012
None	1 (2.8)	1 (2.9)	7 (20.0)	0.001
Alfacalcidol/ Falecalcitriol	10 (27.8)	18 (51.4)	20 (57.1)	
Calcitriol (i.v.)	14 (38.9)	13 (37.1)	5 (14.3)	
Maxacalcitol (i.v.)	11 (30.6)	3 (8.6)	3 (8.6)	
P binder use, n (%)	30 (83.3)	31 (88.6)	29 (82.9)	0.759
None	6 (16.7)	4 (11.4)	6 (17.1)	0.471
Ca containing	3 (8.3)	3 (8.6)	6 (17.1)	
Non-Ca containing	20 (55.6)	22 (62.9)	13 (37.1)	
Both	7 (19.4)	6 (17.1)	10 (28.6)	
Steroid use history, n (%)	5 (13.9)	4 (11.4)	4 (11.4)	0.935
β blocker use, n (%)	18 (50.0)	18 (51.4)	12 (34.3)	0.277
Statin use, n (%)	12 (33.3)	7 (20.0)	9 (25.7)	0.441
Warfarin use, n (%)	0 (0)	4 (11.4)	3 (8.6)	0.130
Bisphosphonate use, n (%)	5 (13.9)	4 (11.4)	5 (14.3)	0.929
Fracture history, n	3	6	6	0.468
Ankle-brachial index	1.10 [1.03, 1.18]	1.09 [1.03, 1.18]	1.10 [1.01, 1.17]	0.819
Alb, g/dL	3.6 [3.3, 3.8]	3.6 [3.4, 3.8]	3.6 [3.4, 3.7]	0.628
cCa, mg/dL	8.9 [8.4, 9.3]	9.2 [8.6, 9.6]	9.1 [8.7, 9.5]	0.109
P, mg/dL	5.6 [4.7, 6.4]	5.3 [4.5, 6.0]	5.0 [4.3, 5.6]	0.054
iPTH, pg/mL	300.1 [227.9, 441.2]	196.7 [129.3, 269.2]	118.8 [66.8, 187.8]	0.0001
25(OH)D, ng/mL	14.0 [11.6, 18.0]	16.6 [12.3, 20.3]	13.3 [9.7, 17.3]	0.075
1, 25(OH)_2_D, pg/mL	10.0 [5.0, 14.5]	15.0 [11.0, 22.0]	16.0 [8.0, 21.0]	0.003
BAP, μg/L	12.7 [9.3, 18.2]	14.3 [10.8, 18.3]	15.0 [11.3, 19.4]	0.343
TRACP-5b, mU/dL	347.5 [239.5, 500.5]	591.0 [339.0, 777.0]	848.0 [567.0, 1060.0]	0.0001

*Abbreviations*: *Alb* Serum albumin, *BAP* Bone-specific alkaline phosphatase, *BMI* Body mass index, *CaSR* Calcium-sensing receptor, *cCa* Corrected Ca, *ESRD* End-stage renal disease, *HD* Hemodialysis, *iPTH* Intact parathyroid hormone, *i.v.* Intravenous, *LMWH* Low-molecular-weight heparin, *P* Phosphorus, *TRACP-5b* Tartrate-resistant acid phosphatase-5b, *VDRA* Vitamin D receptor activator

Values are expressed as the median [interquartile range (IQR)] or as frequencies and percentages [n (%)]

Median age, BMI, plasma iPTH, and serum 1,25(OH)_2_D, TRACP-5b levels; the number of patients receiving CaSR agonists; and the number of patients receiving VDRAs were significantly different among the three categories (Table 2). The types of CaSR agonists and VDRAs used were also significantly different among the three categories.

### Correlation between plasma iPTH and serum TRACP-5b levels

**Fig. 2:**
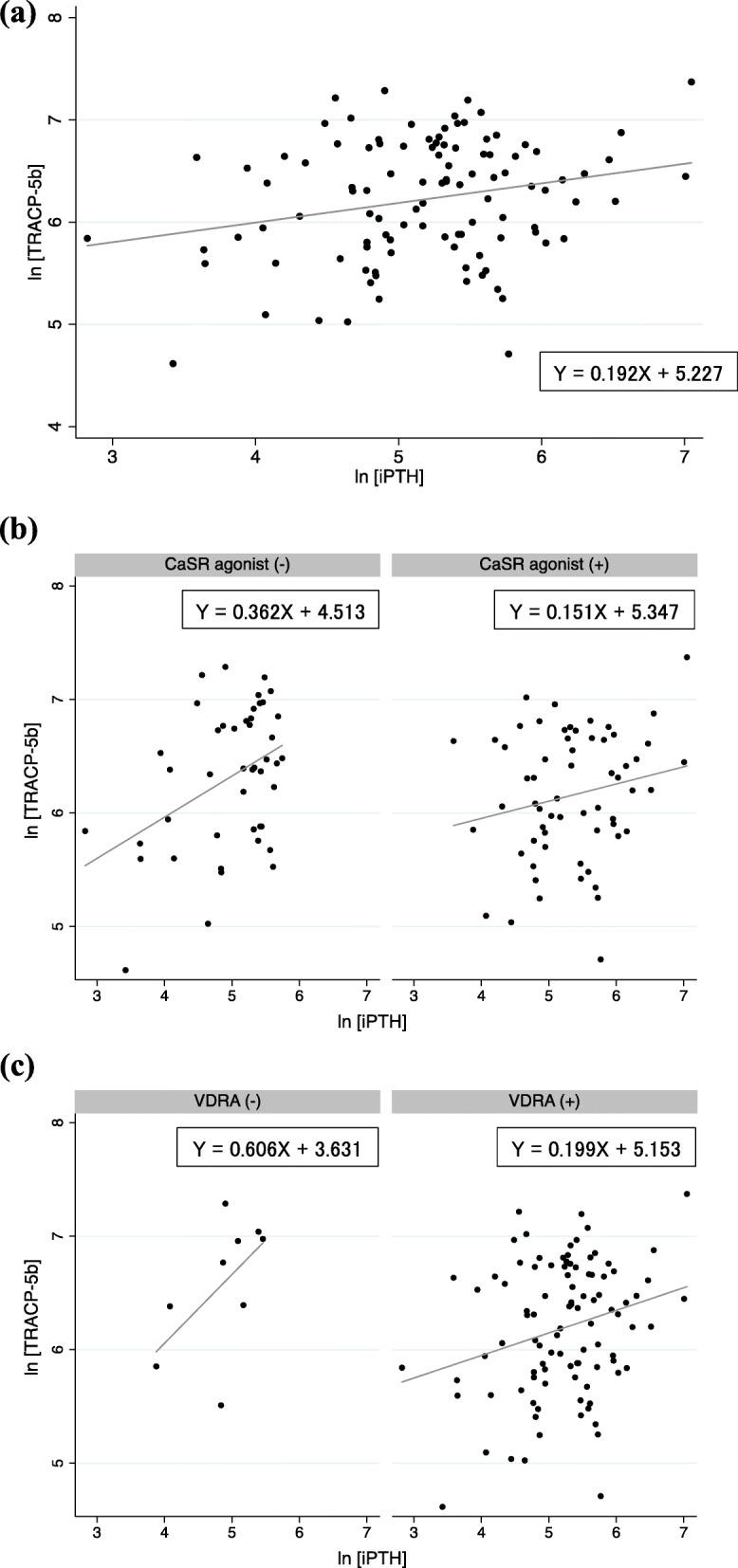
Scatterplots of ln [iPTH] with ln [TRACP-5b] with linear regression prediction lines. The plots depict results in **a** all patients, **b** patients receiving the CaSR agonist, and **c** patients receiving the VDRA. Abbreviations: CaSR, calcium-sensing receptor; iPTH, intact parathyroid hormone; ln, natural logarithm; TRACP-5b, tartrate-resistant acid phosphatase-5b; VDRA, vitamin D receptor activator

Relative to the serum iPTH level, the serum TRACP-5b level of the patients varied as well (adjusted R^2^ = 0.049) (Fig. 2a). Serum TRACP-5b levels showed a weaker correlation with plasma iPTH levels in patients treated with the CaSR agonist than in patients treated without the CaSR agonist (Fig. 2b). Similarly, serum TRACP-5b levels showed a weaker correlation with plasma iPTH levels in patients treated with the VDRA than in patients treated without the VDRA (Fig. 2c).

### Association between CaSR agonists and VDRAs and the TRACP-5b/iPTH ratio

We examined whether CaSR agonist and/or VDRA use was associated with bone responsiveness to PTH. The results of the single linear regression analysis using ln [TRACP-5b/iPTH] as a dependent variable are shown in Table 3. The single linear regression analysis revealed a significant positive correlation between ln [TRACP-5b/iPTH] and age, serum cCa level, and ln [1,25(OH)_2_D] and a significant negative correlation between ln [TRACP-5b/iPTH] and BMI, serum P level, CaSR agonist use, and VDRA use. Moreover, upon dividing the patient cohort according to the types of CaSR agonists and VDRAs, the single linear regression analysis also showed a significant negative correlation between ln [TRACP-5b/iPTH] and cinacalcet/evocalcet use, etelcalcetide use (intravenously, i.v.), calcitriol use (i.v.), and maxacalcitol use (i.v.).

**Table 3:** Single linear regression analysis for ln [TRACP-5b/iPTH]

Variable	Coefficient	***P*** value	95% CI
Age, years	0.018	0.016	0.003, 0.032
Female sex	0.152	0.353	−0.171, 0.476
BMI, kg/m^2^	−0.042	0.026	−0.080, − 0.005
Chronic HD vintage, years	− 0.005	0.639	− 0.027, 0.017
Anticoagulant during HD			
Heparin	(base)	(base)	(base)
LMWH	− 0.359	0.194	−0.905, 0.186
Nafamostat	0.373	0.287	−0.317, 1.063
Cause of ESRD			
Diabetic nephropathy	(base)	(base)	(base)
Nephrosclerosis	−0.041	0.854	−0.479, 0.397
Glomerulonephritis	−0.046	0.831	−0.470, 0.379
Others	0.156	0.516	−0.320, 0.633
Diabetes	−0.096	0.569	−0.431, 0.278
Ischemic heart disease	−0.085	0.647	−0.453, 0.283
Cerebrovascular disease	−0.294	0.171	−0.718, 0.129
CaSR agonist use	−0.532	0.001	−0.840, − 0.225
None	(base)	(base)	(base)
Cinacalcet/ Evocalcet	−0.450	0.004	−0.757, − 0.143
Etelcalcetide (i.v.)	−1.290	< 0.001	− 1.953, − 0.625
VDRA use	− 0.744	0.009	− 1.301, − 0.187
None	(base)	(base)	(base)
Alfacalcidol/ Falecalcitriol	−0.467	0.098	− 1.021, 0.087
Calcitriol (i.v.)	−1.013	0.001	−1.588, − 0.437
Maxacalcitol (i.v.)	− 1.021	0.002	− 1.650, − 0.392
P binder use	− 0.041	0.856	− 0.490, 0.407
None	(base)	(base)	(base)
Ca-containing	0.278	0.380	−0.348, 0.905
Non-Ca containing	−0.166	0.482	−0.632, 0.300
Both	0.090	0.739	−0.444, 0.624
Steroid use history	−0.212	0.391	−0.699, 0.276
β blocker use	−0.165	0.310	−0.486, 0.156
Statin use	−0.129	0.483	−0.492, 0.234
Warfarin use	0.432	0.184	−0.209, 1.073
Bisphosphonate use	−0.120	0.615	−0.594, 0.353
Fracture history	0.185	0.427	−0.274, 0.644
Ankle-brachial index	−0.115	0.831	−1.176. 0.947
Alb, g/dL	−0.251	0.344	−0.774, 0.272
cCa, mg/dL	0.319	0.007	0.090. 0.548
P, mg/dL	−0.173	0.018	−0.316, − 0.031
ln [25(OH)D]	− 0.275	0.208	− 0.705, 0.155
ln [1, 25(OH)_2_D]	0.250	0.044	0.007, 0.493
ln [BAP]	0.195	0.282	−0.163, 0.553

*Abbreviations*: *Alb* Serum albumin, *BAP* Bone-specific alkaline phosphatase, *BMI* Body mass index, *CaSR* Calcium-sensing receptor, *cCa* Corrected Ca, *ESRD* End-stage renal disease, *HD* Hemodialysis, *iPTH* Intact parathyroid hormone, *i.v.* Intravenous, *LMWH* Low-molecular-weight heparin, *ln* Natural logarithm, *P* Phosphorus, *TRACP-5b* Tartrate-resistant acid phosphatase-5b, *VDRA* Vitamin D receptor activator

We next conducted a multiple linear regression analysis with adjustment for age, sex, BMI, and serum Alb level using variables identified as significant in the single linear regression analysis (i.e., CaSR agonist use, VDRA use, serum cCa level, serum P level, and ln [1,25(OH)_2_D]) (Table 4). Model 1 was unadjusted; model 2 was adjusted for age and sex; model 3 was adjusted for age, sex, and BMI; and model 4 was adjusted for age, sex, BMI, and serum Alb level. In all adjusted models, etelcalcetide use (i.v.), calcitriol use (i.v.), and maxacalcitol use (i.v.) exhibited a significant negative correlation with ln [TRACP-5b/iPTH].

**Table 4:** Multiple linear regression analysis for ln [TRACP-5b/iPTH]

Variable	Model 1	Model 2	Model 3	Model 4
Age, years		0.006 [−0.008, 0.021]	0.004 [−0.011, 0.019]	0.007 [− 0.009, 0.023]
Female sex		0.204 [−0.086, 0.494]	0.186 [−0.107, 0.478]	0.190 [− 0.103, 0.484]
BMI, kg/m^2^			−0.019 [− 0.057, 0.019]	−0.020 [− 0.057, 0.018]
Alb, g/dL				0.219 [−0.321, 0.759]
CaSR agonist use				
None	(base)	(base)	(base)	(base)
Cinacalcet/ Evocalcet	−0.329* [− 0.642, − 0.015]	−0.313 [− 0.630, 0.004]	−0.296 [− 0.623, 0.030]	−0.284 [− 0.612, 0.045]
Etelcalcetide (i.v.)	−1.029** [−1.716, − 0.342]	−0.932** [− 1.629, − 0.236]	−0.940** [− 1.639, − 0.240]	−0.886* [− 1.599, − 0.173]
VDRA use				
None	(base)	(base)	(base)	(base)
Alfacalcidol/ Falecalcitriol	−0.349 [− 0.926, 0.236]	−0.344 [− 0.927, 0.239]	−0.299 [− 0.888, 0.290]	−0.305 [− 0.895, 0.285]
Calcitriol (i.v.)	− 0.770** [− 1.340, − 0.200]	−0.815** [− 1.389, − 0.240]	−0.772* [− 1.353, − 0.191]	−0.791** [− 1.374, − 0.207]
Maxacalcitol (i.v.)	−0.749* [− 1.385, − 0.113]	−0.791* [− 1.434, − 0.148]	−0.810* [− 1.456, − 0.164]	−0.831* [− 1.481, − 0.182]
cCa, mg/dL	0.171 [− 0.057, 0.399]	0.117 [− 0.057, 0.398]	0.177 [− 0.051, 0.406]	0.209 [− 0.033, 0.451]
P, mg/dL	−0.123 [− 0.264, 0.017]	−0.113 [− 0.257, 0.029]	−0.095 [− 0.245, 0.054]	−0.097 [− 0.247, 0.053]
ln [1, 25(OH)_2_D]	− 0.156 [− 0.470, 0.158]	−0.207 [− 0.528, 0.113]	−0.207 [− 0.529, 0.115]	−0.213 [− 0.535, 0.110]

*Abbreviations*: *Alb* Serum albumin, *BMI* Body mass index, *CaSR* Calcium-sensing receptor, *cCa* Corrected Ca, *iPTH* Intact parathyroid hormone, *i.v.* Intravenous, *ln* Natural logarithm, *P* Phosphorus, *TRACP-5b* Tartrate-resistant acid phosphatase-5b, *VDRA* Vitamin D receptor activator

Values are expressed as the median [interquartile range (IQR)] or as frequencies and percentages [n (%)]. *P < 0.05, **P < 0.01

Coefficient [95% CI]

Model 1: CaSR agonist use (None, Cinacalcet/Evocalcet, Etelcalcetide), VDRA use (None, Alfacalcidol/ Falecalcitriol, Calcitriol, Maxacalcitol), cCa, P, ln [1, 25(OH)_2_D]

Model 2: CaSR agonist use (None, Cinacalcet/Evocalcet, Etelcalcetide), VDRA use (None, Alfacalcidol/ Falecalcitriol, Calcitriol, Maxacalcitol), cCa, P, ln [1, 25(OH)_2_D], Age, Sex

Model 3: CaSR agonist use (None, Cinacalcet/Evocalcet, Etelcalcetide), VDRA use (None, Alfacalcidol/ Falecalcitriol, Calcitriol, Maxacalcitol), cCa, P, ln [1, 25(OH)_2_D], Age, Sex, BMI

Model 4: CaSR agonist use (None, Cinacalcet/Evocalcet, Etelcalcetide), VDRA use (None, Alfacalcidol/ Falecalcitriol, Calcitriol, Maxacalcitol), cCa, P, ln [1, 25(OH)_2_D], Age, Sex, BMI, Alb

Additionally, in order to uncover the association between mineral metabolism and ln [TRACP-5b/iPTH], we performed an additional multiple linear regression analysis for ln [TRACP-5b/iPTH] only using variables identified as significant in the single linear regression analyses, such as age, BMI, serum cCa, P levels, and ln [1,25(OH)_2_D], shown in Table 3. Only the serum cCa level showed a significant positive association with ln [TRACP-5b/iPTH] (Table 5).

**Table 5:** Multiple linear regression analysis on mineral metabolism for ln [TRACP-5b/iPTH]

Variable	Coefficient	P value	95% CI
Age	0.009	0.256	−0.007, 0.025
BMI	−0.026	0.180	−0.065, 0.012
cCa	0.243	0.044	0.007, 0.480
P	−0.080	0.307	−0.235, 0.075
ln [1,25(OH)_2_D]	0.078	0.563	−0.190, 0.347

*Abbreviations*: *BMI* Body mass index, *cCa* Corrected Ca, *ln* Natural logarithm, *P* Phosphorus

## Discussion

To the best of our knowledge, this study is the first report demonstrating that bone responsiveness to PTH is significantly negatively associated with the intravenous administration of CaSR agonist and/or VDRA in patients undergoing chronic HD. High bone turnover is the central pathophysiology in SHPT, which is related to an increased risk of fracture [1]. Therefore, the management of SHPT is expected to improve bone metabolism and reduce the risk of fractures. In this aspect, the management goal of iPTH levels remains an important issue. Based on the relationship between the iPTH level and mortality risk in observational studies [10], the Kidney Disease: Improving Global Outcomes (KDIGO) guideline for CKD-MBD in 2009 suggested revising the management goal of iPTH levels to 2–9 times (130–585 pg/mL) of the normal upper limits [11]. Although the 2012 guidelines of the Japanese Society for Dialysis Therapy (JSDT) also suggested target iPTH levels of 60–240 pg/mL based on mortality [12], the target levels of JSDT are close to the target iPTH levels of 150–300 pg/mL proposed by the K/DOQI guidelines in 2003 based on the correlation of iPTH levels with bone turnover by bone morphometry [13].

Bone loss observed in patients receiving chronic HD affected cortical bone mineral density and thickness, which are correlated with high PTH levels and chronic HD vintage [14]. Moreover, PTH levels a couple of times higher than normal levels are considered necessary to maintain normal bone turnover in patients with chronic kidney disease (CKD); low bone responsiveness to PTH is also observed as CKD progresses [15]. Sprague et al. reported the diagnostic accuracy of bone-turnover markers and bone histology in patients on dialysis (almost all the patients were on HD) [16], demonstrating that PTH is still the best tool for the non-invasive assessment of bone turnover in clinical practice; however, TRACP-5b was not assessed in this study.

The serum TRACP-5b activity is correlated with bone resorption marker levels and osteoclast numbers [17, 18]. Although literature on TRACP-5b in patients on dialysis is scarce, the TRACP-5b level was found to be correlated with most histomorphometric and histodynamic parameters [19]. Additionally, TRACP-5b is the only bone resorption marker not cleared by the kidneys and is an ideal marker for assessing bone resorption in patients on chronic HD [20]. The intra-individual coefficient of variation of TRACP-5b in patients receiving chronic HD is quite low (8.3%) [21].

For the above reasons, to investigate bone responsiveness to PTH, we retrospectively assessed the TRACP-5b/iPTH ratio in patients receiving chronic HD. In the present study, the median patient age was 68 years, 42.5% patients were female (all postmenopausal), 35.8% had type 2 diabetes, and diabetic nephropathy was the cause of ESRD in approximately one-third of the patients (Table 1). In the laboratory tests, the median serum cCa, P, and plasma iPTH levels were 9.1 mg/dL, 5.4 mg/dL, and 199.1 pg/mL, respectively, all of which were in the recommended ranges based on the Japanese CKD-MBD guideline [12]. The median TRACP-5b level was 559 mU/dL (Table 1). Including patients with CKD, bone responsiveness to PTH increases in the following order: men < premenopausal women < postmenopausal women [22]. In the present study, there was no significant difference in the percentage of female patients among the three categories (Table 2). Although bone responsiveness to PTH decreases in patients on chronic HD and those with diabetes [23], the percentage of patients with diabetes was not significantly different among the three categories (Table 2). The median serum 1,25(OH)_2_D level was significantly different among the three TRACP-5b/iPTH categories (Table 2). Disturbed mineral metabolism, i.e., higher serum cCa and P and lower serum 1,25(OH)_2_D levels, was associated with reduced bone responsiveness to PTH in the single linear regression analyses. In an additional multiple linear regression analysis performed to uncover the association between mineral metabolism and ln [TRACP-5b/iPTH] only using the variables identified as significant in the single linear regression analyses (age, BMI, serum cCa, P levels, and ln [1,25(OH)_2_D]), only the serum cCa level exhibited a significant positive association with ln [TRACP-5b/iPTH] (Table 5). However, from a clinical point of view, higher serum P and lower serum 1,25(OH)_2_D levels could lead to higher plasma iPTH levels, resulting in lower TRACP-5b/iPTH ratios. Moreover, the decreased bone responsiveness to PTH in younger patients could be because higher P intake can lead to a high PTH level, resulting in a lower TRACP-5b/iPTH ratio. On the contrary, in postmenopausal and/or lean older patients with low protein intake and/or exercise, serum TRACP-5b levels might increase, leading to a higher TRACP-5b/iPTH ratio.

In clinical practice, we prescribe a CaSR agonist and/or a VDRA to decrease high iPTH levels; therefore, the iPTH level might be related to the serum 1,25(OH)_2_D level in each category. Interestingly, the types of CaSR agonists and VDRAs used were significantly different among the three categories (Table 2). Upon conducting multiple linear regression analysis incorporating variables identified as significant in the single linear regression analysis (Table 3), a significant negative correlation was found between the TRACP-5b/iPTH ratio and the intravenous administration of a CaSR agonist (etelcalcetide) and/or a VDRA (calcitriol or maxacalcitol) in all the adjusted models (Table 4).

Some reports mention the potential effects of CaSR agonists on bone responsiveness to PTH. CaSR agonists may directly affect bone cells because CaSR is expressed on bone tissue [24]. Activation of CaSR in osteocytes and a high extracellular Ca level seem to exert osteogenic effects by inducing osteoblast differentiation and osteoclast apoptosis [25]. It has been reported that strontium, a calcimimetic, stimulates CaSR, promotes the differentiation of preosteoblasts into osteoblasts, promotes bone formation, and inhibits the action of osteoclast differentiation factors by secreting osteoprotegerin (OPG) from osteoblasts, which can lead to the inhibition of bone resorption by preventing the RANKL-mediated differentiation of preosteoblasts into osteoclasts [26]. Díaz-Tocados et al. [27] first reported that AMG 641, an allosteric CaSR agonist, exerted a direct anabolic effect on the bone through activation of the CaSR in bone cells of a uremic rat model. In nephrectomized rats with parathyroidectomy and PTH infusion, the CaSR agonist increased the bone turnover at low PTH levels but not at high PTH levels, suggesting that SHPT reduces the direct effect of CaSR agonists on the bone. Since CaSR agonists and VDRAs were administered to our patients with SHPT, the reduced bone responsiveness to PTH observed in these patients (indicated by a steep coefficient in Fig. 2b and c) is consistent with the results reported previously.

In the participating centers of the present study, the choice of a CaSR agonist for the treatment for SHPT mainly depended on (1) whether patients could take it orally, (2) the degree of gastrointestinal symptoms caused by an oral CaSR agonist (cinacalcet), and (3) whether high PTH levels could be controlled by an oral CaSR agonist, in this order. In the present study, only the use of etelcalcetide showed a significant negative correlation with the TRACP-5b/iPTH ratio among CaSR agonists, although the number of patients receiving etelcalcetide was small (Table 4). Etelcalcetide, a synthetic peptide CaSR agonist, is the first intravenous formulation with a mechanism of action similar to that of cinacalcet [28]; etelcalcetide was found to be superior to cinacalcet in the reduction of iPTH levels among patients on chronic HD with SHPT with iPTH levels greater than 500 pg/mL [29]. Notably, etelcalcetide and cinacalcet show differences in terms of pharmacokinetics. Since the half-life of cinacalcet is approximately 30 h, PTH levels in patients receiving cinacalcet show oscillations [30]. However, the half-life of etelcalcetide is as long as 3–5 days [31]; thus, etelcalcetide keeps PTH secretion suppressed and may not cause PTH level oscillations in patients with SHPT. This difference in pharmacokinetics among CaSR agonists may also influence bone responsiveness to PTH.

The introduction of intravenous calcitriol was associated with marked reductions in PTH levels and bone turnover [32], and a study reported improvement in high-turnover bone lesions by calcitriol in chronic HD patients [33]. There are also some reports on the potential effects of VDRA on bone responsiveness to PTH. By acting on osteoblasts, VDRA produces OPG, which neutralizes RANKL, suppresses osteoclast production and activity, and finally induces osteoclast apoptosis [34]. There are reports indicating that calcitriol downregulates PTH/PTHrP receptor gene expression in osteoblast-like cells [35] and that intracellular cAMP levels increase in response to PTH in osteoblasts, but this increase is suppressed by treatment with 1,25(OH)_2_D [36]. Kikuta et al. reported that VDRAs significantly suppress the expression of sphingosine-1-phosphate receptor 2 in circulating osteoclasts and inhibit bone resorption by osteoclasts through monocyte progenitor cells migrating from the bone to blood [37].

In addition to the use of the intravenous CaSR agonist in the present study, only the use of the intravenous VDRAs exhibited a significant negative correlation with the TRACP-5b/iPTH level among VDRAs (Table 4). In a prospective randomized study, the effects of oral and intravenous pulse calcitriol on serum levels of IL-1 beta and IL-6 and bone-resorptive cytokines implicated for changes in bone remodeling were compared in patients on chronic HD [38]. Despite there being no difference in serum PTH levels after 6 months, intravenous calcitriol therapy caused a greater increase in bone mineral densities and a greater decrease in serum IL-6 levels than oral calcitriol therapy; this suggested that compared to oral therapy, intravenous calcitriol treatment exerted a superior effect on bone remodeling by influencing the levels of bone-resorptive cytokines, beyond its suppressive effect on iPTH. The iPTH assay was used to measure the total length of PTH, consisting of 84 amino groups, simultaneously including a large C-PTH fragment, such as PTH (7–84) (cyclase inhibitory PTH, CIP) [39]. Furthermore, PTH (7–84) was shown to have an antagonistic effect on PTH (1–84) (cyclase activating PTH, CAP) [38]. Of note, intravenous VDRA treatment was shown to reduce the PTH (1–84)/large C fragments ratio in patients on chronic HD [40], which could reduce bone responsiveness to PTH. Indeed, maxacalcitol is as effective as calcitriol in suppressing PTH in patients on chronic HD [41], but a report demonstrated that intravenous maxacalcitol therapy in chronic HD patients markedly improved bone histology, without a decrease in PTH levels [42].

The present study has several strengths. This is the first report on the association between CaSR agonist and/or VDRA use and bone responsiveness to PTH in a chronic HD population. In clinical practice, the commonly utilized bone-turnover markers in CKD are BAP and iPTH; however, we used TRACP-5b as a specific biomarker of osteoclast number and activity, as it is not influenced by renal function and its level was reported to be correlated with most histomorphometric and histodynamic parameters [19]. We also used the means of six consecutive values of plasma iPTH levels measured once per month, prior to measuring the serum TRACP-5b level, to avoid influence by a single iPTH level. We created the TRACP-5b/iPTH ratio as an index of bone responsiveness to PTH. Indeed, the major mechanisms underlying the lowering of PTH levels are the inhibition of PTH secretion by CaSR agonists and the suppression of PTH synthesis by VDRAs in the parathyroid glands. However, the present study suggests that HD patients with low TRACP-5b/iPTH ratios are representative of those with bone resistance (decreased bone responsiveness) to PTH, probably associated with severe SHPT, which requires treatment with CaSR agonists and/or VDRAs, as reported by Díaz-Tocados et al. [27]. Therefore, the TRACP-5b/iPTH ratio could serve as an index of the bone responsiveness to PTH in patients on chronic HD in clinical practice.

Several limitations should also be considered while interpreting or generalizing the present findings. First, this was a retrospective, cross-sectional study that used limited information available in each patient’s electronic health record; thus, we could not confirm a causal relationship between the intravenous use of a CaSR agonist and/or VDRA and bone responsiveness to PTH expressed by the TRACP-5b/iPTH ratio. In fact, CaSR agonists and/or VDRAs are prescribed to lower high iPTH levels in clinical practice, which means that there might or might not be differences in the backgrounds of patients subjected to intravenous administration of the two agents. Second, we have addressed neither the dosing period nor the dosage of these drugs. Third, there is a lack of data regarding whole PTH, FGF-23 levels, and bone histomorphometry; thus, the findings of this study do not support further speculation regarding the causal relationship between the intravenous use of a CaSR agonist and/or VDRA and bone responsiveness to PTH. Fourth, the number of patients included in the analysis was relatively small, implying that the statistical significance of the results was low. Finally, this study was conducted only in Japanese SHPT patients undergoing chronic HD whose iPTH levels were controlled to be lower than those of patients in other regions, based on the Japanese CKD-MBD guideline [12].

## Conclusions

The present study indicates that bone responsiveness to PTH not only varies but also is negatively associated with the intravenous administration of CaSR agonists and/or VDRAs in patients undergoing chronic HD.

## Abbreviations

SHPT: Secondary hyperparathyroidism
HD: Hemodialysis
PTH: Parathyroid hormone
RANKL: Receptor activator of nuclear factor-kappa B ligand
TRACP-5b: Tartrate-resistant acid phosphatase 5b
CaSR: Calcium-sensing receptor
VDRA: Vitamin D receptor activator
iPTH: Intact parathyroid hormone
IRB: Institutional Review Board
Alb: Albumin
Ca: Calcium
P: Phosphorus
BAP: Bone-specific alkaline phosphatase
25(OH)D: 25-hydroxyvitamin D
1,25(OH)_2_D: 1,25-dihydroxycholecalciferol
cCa: Corrected calcium
CLEIA: Chemiluminescent enzyme immunoassay
EIA: Enzyme immunoassay
ECLIA: Electrochemiluminescence assay
CLIA: Chemiluminescence assay
RIA: Radioimmunoassay
ABI: Ankle-brachial index
BMI: Body mass index
IQR: Interquartile range
CI: Confidence interval
ln: Natural logarithm
i.v.: Intravenously
KDIGO: the Kidney Disease: Improving Global Outcomes
JSDT: The Japanese Society for Dialysis Therapy
CKD: Chronic kidney disease
OPG: Osteoprotegerin
CIP: Cyclase inhibitory PTH
CAP: Cyclase activating PTH
